# Multi-step FRET systems based on discrete supramolecular assemblies

**DOI:** 10.1038/s42004-024-01175-6

**Published:** 2024-04-18

**Authors:** Dengli Chen, Tangxin Xiao, Éric Monflier, Leyong Wang

**Affiliations:** 1 School of Petrochemical Engineering, Changzhou University, Changzhou, China; 2 Unité de Catalyse et Chimie du Solide (UCCS), Faculté des Sciences Jean Perrin, Univ. Artois, CNRS, Centrale Lille, Univ. Lille, UMR 8181, Lens, France; 3 Jiangsu Key Laboratory of Advanced Organic Materials, School of Chemistry and Chemical Engineering, Nanjing University, Nanjing, China

**Keywords:** Supramolecular chemistry, Materials for energy and catalysis, Light harvesting

## Abstract

Fluorescence resonance energy transfer (FRET) from the excited state of the donor to the ground state of the acceptor is one of the most important fluorescence mechanisms and has wide applications in light-harvesting systems, light-mediated therapy, bioimaging, optoelectronic devices, and information security fields. The phenomenon of sequential energy transfer in natural photosynthetic systems provides great inspiration for scientists to make full use of light energy. In recent years, discrete supramolecular assemblies (DSAs) have been successively constructed to incorporate donor and multiple acceptors, and to achieve multi-step FRET between them. This perspective describes recent advances in the fabrication and application of DSAs with multi-step FRET. These DSAs are categorized based on the non-covalent scaffolds, such as amphiphilic nanoparticles, host-guest assemblies, metal-coordination scaffolds, and biomolecular scaffolds. This perspective will also outline opportunities and future challenges in this research area.

## Abstract (web summary)

Fluorescence resonance energy transfer (FRET) is one of the most important fluorescence mechanisms, with multi-step FRET systems enabling sequential energy transfer as seen in natural photosynthetic systems. Here, the authors review recent progress in exploiting discrete supramolecular assemblies to achieve multi-step FRET between donors and multiple acceptors.

## Introduction

Fluorescence resonance energy transfer (FRET) has received increasing attention by virtue of its important role in natural photosynthetic system and monitoring interactions between biomolecules^1–3^. FRET is non-radiative energy transfer that occurs by dipole-dipole coupling from the excited state of the donor (D) to the ground state of the acceptor (A)^4^. There is a strong correlation between the D–A distance and FRET efficiency, which makes FRET technology widely used in the following fields: light harvesting^5–9^, fluorescence sensing/imaging^10,11^, optoelectronic devices^12,13^ and determination of inter(bio)molecular interaction^1,14^. Nowadays, the development of FRET systems has become one of the most promising topics in the fields of analytical chemistry, chemical biology and materials science^10,15–17^. An efficient FRET process must have at least the following prerequisites: (1) since FRET efficiency is inversely proportional to the sixth power of the D-A spacing, the donor-acceptor distance should be within 10 nm; (2) the emission spectrum of the donor should have a good overlap with the absorption spectrum of the acceptor^4^.

**Fig. 1:**
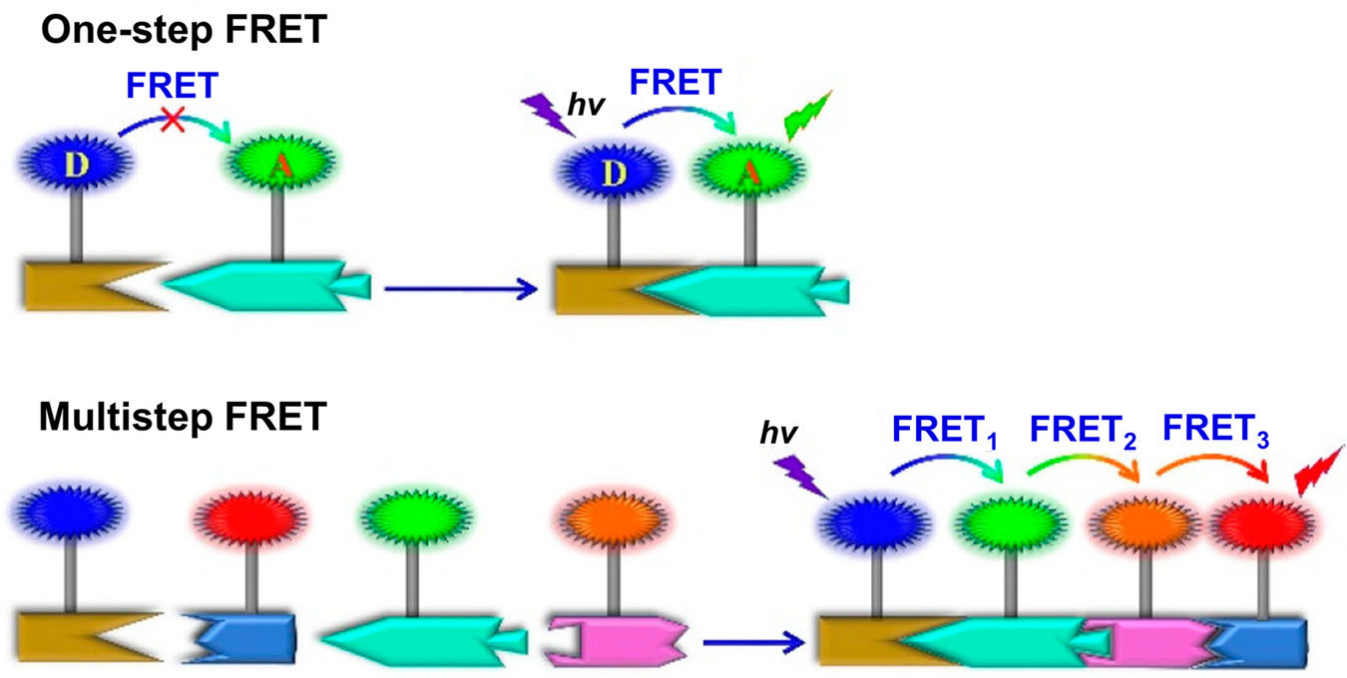
One- and multi-step FRET systems. Cartoon illustration of one-step FRET and multi-step FRET. Reprinted with permission from ref. ^18^, Copyright 2021 American Chemical Society.

It is worth noting that multi-step FRET systems containing multiple types of chromophores have stimulated a lot of interest in recent years. For example, a two-step FRET system usually has a kind of energy donor and two kinds of energy acceptors, the relay acceptor and the final acceptor. The donor chromophores act as a light collecting antenna, capturing the excitation energy and transferring it to the final acceptor *via* the relay acceptor. The multi-step FRET systems have several advantages over one-step FRET systems (Fig. 1)^18^: (1) large Stokes shifts can be achieved even without spectral overlap between donor and final acceptor; (2) long-range (>10 nm) energy transfer can be achieved; (3) the luminescent color of the system has a wider adjustment range; (4) it is a better mimicking of multi-step energy transfer in natural light-harvesting systems (LHS). On this basis, a series of multi-step FRET systems have been reported in recent decades^19–22^. However, many of these systems are based on covalently bonding multiple fluorophores to polymeric or biomolecular backbones, which faces tedious synthesis, large reagent consumption, and low yields.

Supramolecular chemistry is “chemistry beyond the molecule”, focusing on molecular recognition and discrete nanoassemblies driven by noncovalent interactions^23,24^. These noncovalent interactions include hydrogen bonding, π–π interactions, metal–ligand coordination, hydrophobic forces, and electrostatic interaction etc.^25,26^. In contrast to a covalent synthetic approach, multi-step FRET systems self-assembled by noncovalent interactions can not only avoid tedious synthesis and laborious purification, but also endow the system with stimuli-responsive properties on account of the dynamic nature of noncovalent interactions^27–29^. Furthermore, the photophysical properties of the supramolecular multi-step FRET materials could be easily tuned by controlling the donor-acceptor ratios. It is worth noting that the formation of supramolecular assemblies restricts the motion of fluorophores, greatly inhibiting the non-radiative pathway of excited-state donors to obtain efficient emission, while creating a confined environment for the development of sequential FRET systems. For supramolecular sequential FRET systems, the following points should be noted in the selection of energy acceptors: (1) the acceptors could be well co-assembled into the energy transfer nanoplatform; (2) there is good spectral overlap between donors and acceptors at each level of transfer; (3) each acceptor has good antenna effect.

In this perspective, we summarize recent advances in the construction and application of multi-step FRET systems based on discrete supramolecular assemblies (DSAs). Specifically, we discuss the construction of these FRET systems based on different non-covalent scaffolds, such as amphiphile-based nanoparticles, host-guest assemblies, metal-coordination scaffolds, and biomolecular scaffolds. We also discuss their potential applications in information encryption, bio-imaging, photocatalysis, temperature sensing, reactive oxygen species generation, and tunable emission. Finally, we provide our perspectives on the challenges and potential future development directions of DSA-based multi-step FRET systems, providing a reference for the design of functional multi-step FRET systems to promote cross-integration development in related fields.

## Multi-step FRET systems constructed by fluorophores and amphiphiles

Discrete supramolecular nanoparticles assembled from amphiphilic molecules in water are good platforms for constructing FRET systems^30–32^. The donor fluorophores can be loaded into nanoparticles in the following two ways: (1) integrating the fluorophore group as a hydrophobic moiety into the amphiphilic molecule, (2) using additional surfactants to encapsulate the hydrophobic fluorescent molecule. With further loading of multiple types of acceptor molecules *via* non-covalent interactions, the donor and acceptor fluorophores are simultaneously encapsulated in nanometer-sized assemblies, which is conducive to sequential energy transfer. It is worth mentioning that in order to avoid fluorescence quenching in nanoaggregates, aggregation-induced emission (AIE) fluorophores^33,34^ are often used as donor molecules^35–37^. The luminescence mechanism of AIE is generally considered to be the inhibition of non-radiative pathways caused by the restriction of intramolecular motion (RIM). Therefore, in order to further enhance the fluorescence properties of AIE molecules, further strategies, such as macrocyclization or supramolecular polymerization, are usually adopted to enhance the effect of AIE.

**Fig. 2:**
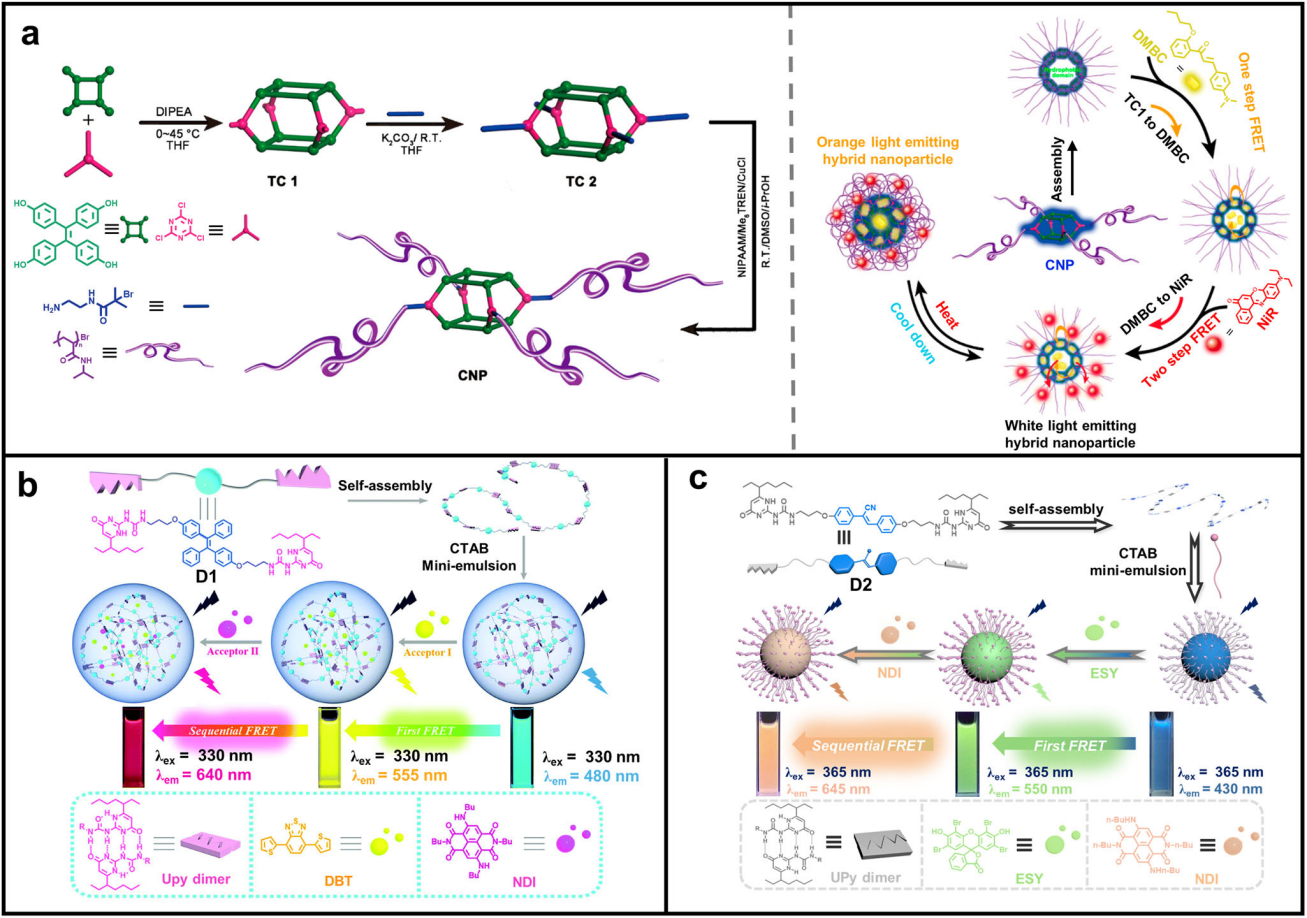
Sequential FRET system constructed by fluorophores and surfactants. **a** Graphical representation of synthesis of cage-based poly(*N*-isopropylacrylamide) polymer (**CNP**) and graphical illustration of **CNP** assembly into hybrid nanoparticle for cascade FRET. Reprinted with permission from ref. ^38^, Copyright 2020 American Chemical Society. **b** Schematic illustration of the construction of a sequential energy-transfer artificial LHS from **D1,** **DBT**, and **NDI**. Reprinted with permission from ref. ^48^, copyright 2020 Royal Society of Chemistry. **c** Cartoon representation of the fabrication of a two-step energy transfer artificial LHS from **D2,** **ESY**, and **NDI**. Reprinted with permission from ref. ^49^, copyright 2024 Elsevier.

In order to prepare a fluorescent probe for temperature sensing in living cells, Zhang, Tang, and co-workers constructed a cascade FRET system based on tetraphenylethylene (TPE) cages (Fig. 2a)^38^. TPE is a well-known AIE fluorophore. The poly(*N*-isopropylacrylamide) (PNIPAM)-decorated amphiphilic cage **CNP** was synthesized by atom transfer radical polymerization (ATRP). The resultant **CNP** self-assembled into blue emissive nanoparticles in water. By co-assembling with other two acceptors, 4-dimethylamino-2’-butoxychalcone (**DMBC**) and Nile Red (**NiR**), a cascade FRET system with tricolor fluorescence was achieved. With the addition of **DMBC**, the emission intensity of **CNP** gradually decreased. Similarly, when different amounts of **NiR** were added, the fluorescence intensity of **DMBC** also gradually decreased. These observations confirmed the occurrence of efficient cascade FERT from **CNP** to **DMBA** to **NiR**. Therefore, by adding different doses of **DMBC** and **NiR** in **CNP** solution (0.5 mg/mL), the fluorescence color of hybrid nanoparticles can be adjusted to full color. Notably, white-light emission was achieved in this hybrid nanoparticle system when **CNP**:**DMBA**:**NiR** = 1:6.40 × 10^−3^:2.24 × 10^−3^ (mass ratio). Interestingly, the thermo-responsive PNIPAM chains further endow the nanoparticles with lower critical solution temperature (LCST) behavior. As a result, the white-light emission solution could be tuned to orange emission when heated to 45 °C and changed back to white-light emission after cooling down. Finally, the authors successfully applied such white-light emission hybrid nanoparticles for temperature sensing in living cells. Therefore, the authors synthesized a multi-step FRET-type fluorescent probe through self-assembly to sense intracellular temperature through fluorescence color changes. This technology not only has high resolution, but also avoids the tedious synthesis process. It has great potential in future biological temperature sensing.

As mentioned above, another efficient strategy to promote AIE is supramolecular polymerization, which is driven by non-covalent interactions to assemble building blocks together. On this basis, Xiao and co-workers employed quadruple hydrogen-bond interaction generated by ureidopyrimidinone (UPy)^39–44^ group to develop several artificial LHSs based on one-step FRET process^45–47^. In a follow-up work, they further constructed a cascade FRET system by using a TPE-bridged ditopic UPy monomer **D1** as energy donor and two dyes, **DBT** and **NDI**, as the first and second acceptors (Fig. 2b)^48^. In aqueous solution, **D1** can self-assemble into supramolecular polymeric nanoparticles with the assistance of cetyltrimethyl ammonium bromide (CTAB) as surfactant to disperse them. As a result, a bright cyan-emission solution was obtained. Upon the addition of the first acceptor **DBT**, emission color of the solution gradually changed to yellow, and further turned into red in the presence of **NDI**. This cascade energy transfer process was verified by both steady-state and transient fluorescence spectroscopy. Notably, this cascade light-harvesting system exhibits a high antenna effect value of 63 when the molar ratio of **D1**:**DBT**:**NDI** = 1250:25:1. This system based on supramolecular polymeric nanoparticles shows great potential in developing dynamic fluorescent materials. Very recently, they further use such mini-emulsion method to build another two-step FRET system based on supramolecular polymeric nanoparticles (Fig. 2c)^49^. In this work, they designed and synthesized a new energy donor **D2**, which contains a cyanostilbene core flanked by two UPy groups. By co-assembling the first acceptor Eosin Y (**ESY**) and the second acceptor **NDI** into the nanoparticles, an efficient sequential LHS could be fabricated. The triangular emission region in CIE (Commission Internationale de l’Eclairage) diagram formed by **D2**-**ESY** and **D2-ESY-NDI** provides the possibility to develop white-light emission materials. As a result, a white-light emission was obtained when the molar ratio of **D2/ESY/NDI** = 1000/5/1. The color coordinate was calculated to be (0.31, 0.33), which is very close to the pure white point (0.33, 0.33). Therefore, tunable emission including a white-light emission was successfully realized in this work.

## Multi-step FRET systems driven by macrocyclic host–guest interactions

Macrocyclic host-guest complexation is one of the most important supramolecular interactions^50–53^. A series of macrocyclic hosts are suitable for host-guest interactions, such as crown ethers^54,55^, cyclodextrins (CD)^56–58^, cucurbit[n]urils^59–62^, calix[n]arenes^63–65^, and pillar[n]arenes^66–68^. In the last decade, a number of one-step FRET systems have been constructed based on macrocyclic host-guest interactions^69–80^. For example, Liu and co-workers reported an efficient FRET system based on the host-guest interaction of cyclodextrin in 2010^81^. In addition, they further developed an artificial supramolecular LHS with an ultrahigh antenna effect based on sulfato-*β*-CD and an AIE guest^82^. In recent years, several sequential FRET systems were constructed by host-guest interactions for different applications, such as photocatalysis, photoluminescence, and reactive oxygen species (ROS) generation.

**Fig. 3:**
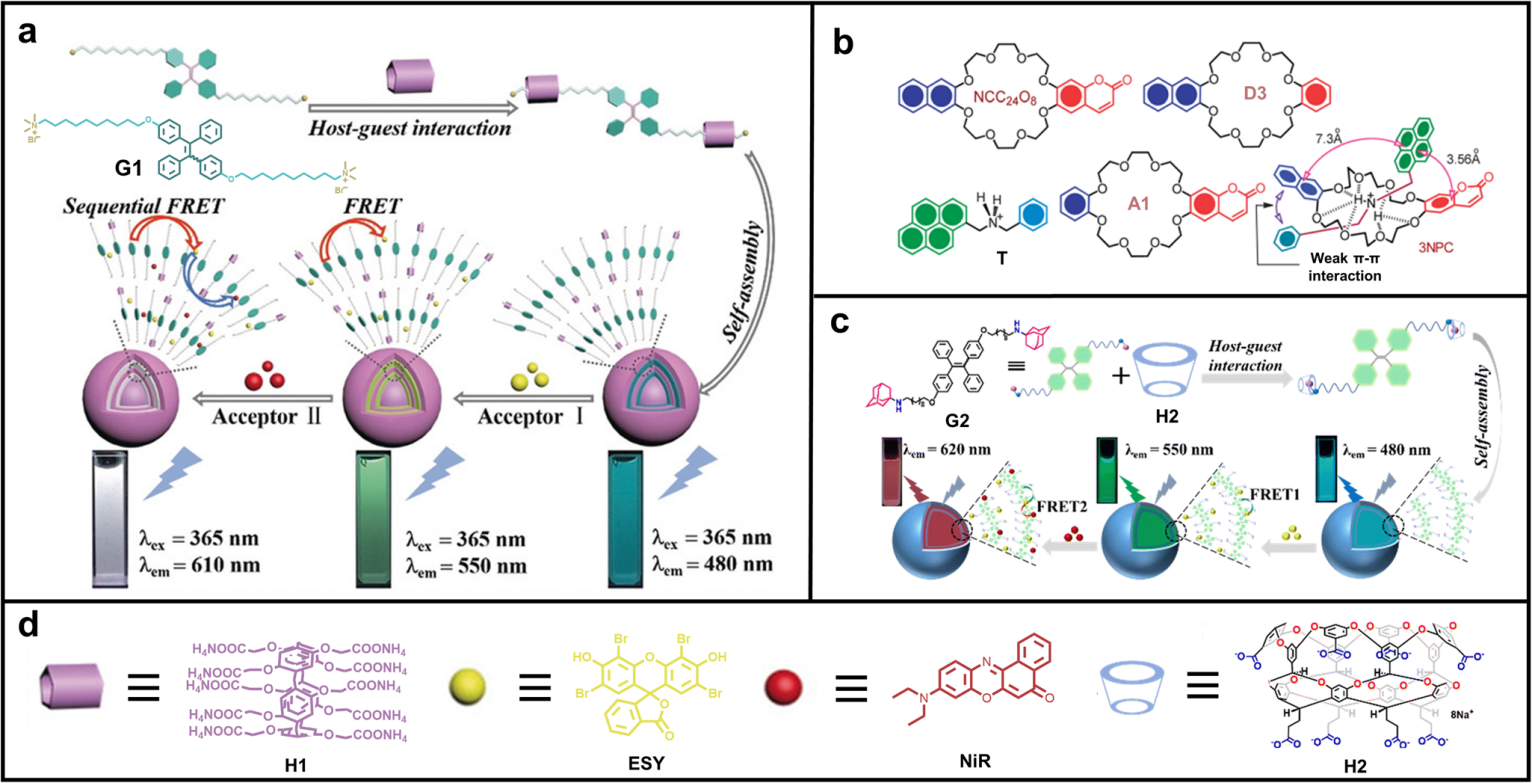
Multi-step FRET systems constructed by macrocyclic host–guest interactions. **a** Illustration of the self-assembly of pillar[5]arene-based aqueous LHS with two-step sequential energy transfer. Reprinted with permission from ref. ^85^, Copyright 2020 Wiley. **b** Molecular structures of crown ether-based host molecules (**NCC**_**24**_**O**_**8**_, D and A), the secondary ammonium ion derivative as guest (**T**), and the proposed conformation of the host–guest pseudorotaxane assembly in solution. Reprinted with permission from ref. ^92^, copyright 2013 Royal Society of Chemistry. **c** Schematic illustration of a cavitand-based supramolecular artificial LHS with sequential energy transfer in aqueous solution. Reprinted with permission from ref. ^96^, copyright 2023 Royal Society of Chemistry. **d** Chemical structures of **H1,** **ESY,** **NiR**, and **H2**.

Pillar[n]arenes are macrocyclic molecules containing dialkoxybenzene groups connected by methylene groups at para-positions^83,84^. The symmetrical pillar structure of pillar[n]arenes endows them with good guest-binding capability. In 2020, Hu, Wang and co-workers reported a LHS with two-step FRET based on pillar[5]arene (Fig. 3a)^85^. They synthesized a TPE-derived bola-type guest molecule **G1** and a water-soluble pillar[5]arene **H1** as the host. The host–guest complexation between **G1** and **H1** in water afforded supra-amphiphiles, which further self-assembled into nanoparticles. The size and morphology of the nanoparticles were characterized by dynamic light scattering (DLS) and transmission electron microscope (TEM), which showed spherical shape with diameter of ca. 180 nm. The commercially available dyes **ESY** and **NiR** were successively co-assembled into the nanoparticles by hydrophobic interaction. As a result, an efficient two-step FRET system was obtained in aqueous media. This system also showed white-light emission when the molar ratio of **G1**:**ESY**:**NiR** = 100:5:2. The CIE coordinate of this white-light emission was calculated to be (0.33, 0.33), which is in accordance with the pure white point. The energy-transfer efficiency (Φ_ET_) of the first-step FRET (**H1**⊃**G1** → **ESY**) was 92.45%, and 74.78% for the second-step FRET (**ESY** → **NiR**), indicating the successful construction of an efficient LHS with cascade energy transfer. It is noteworthy that the authors used this cascade FRET nanoparticles for photocatalysis of dehalogenation. The results showed that the yield was increased from 31% (only **ESY** + **NiR**) to 96%. This is an excellent example of converting light energy into chemical energy and is a deep simulation of natural photosynthesis.

In a follow-up investigation, Hu, Wang and co-workers further constructed a sequential LHS based on pillar[5]arene by changing the TPE unit to a cyanostilbene group and using **DBT** and **NiR** as energy acceptors^86^. This new system exhibited a high antenna effect of 47.8 for the first-step FRET and 20.1 for the second-step FRET. In 2020, Zhang, Liu and co-workers fabricated a cascade FRET system based on water soluble pillar[5]arene and a TPE-derived guest. The authors used sulfonated aluminum phthalocyanine (**AlPcS**_**4**_) as the final acceptor and sulforhodamine 101 (**SR101**) as the relay acceptor. It achieved a large Stokes shift of 340 nm owing to the multiple energy transfer steps from the donor to **SR101** and then to **AlPcS**_**4**_^87^. In 2022, Han, Xing and co-workers also reported a sequential FRET system based on pillar[5]arene and a cyano-substituted *p*-phenylenevinylene guest molecule (**PPTA**)^88^. The **PPTA** molecule plays dual role of both guest and energy donor. In this case, the authors also chose **ESY** and **NiR** as the energy acceptors due to their well-matched photophysical properties with the donor. After completing the construction of this system, they further successfully used it as a photocatalyst to catalyze aerobic cross-dehydrogenation coupling reactions. Moreover, some other sequential energy transfer systems based on pillar[5]arenes were further constructed recently for different applications, including photocatalysis, white-light emission, and ROS generation etc.^89–91^.

Crown ethers are cyclic oligomers of ethylene oxide, which show good host-guest binding abilities towards organic cations, such as ammonium and pyridinium salts^54^. Self-assembly of multiple chromophores in a crown ether-based pseudorotaxane is a promising way for sequential FRET with minimum loss in energy transfer processes. The host-guest complexation can shorten the distance between chromophores and promote FRET efficiency. In 2013, Das and co-workers reported a trichromophoric pseudo[2]rotaxane (**3NPC**) that achieved a two-step FRET based on the complexation of a 24-crown-8 derivative (**NCC**_**24**_**O**_**8**_) and a secondary ammonium ion derivative (**T**) (Fig. 3b)^92^. **NCC**_**24**_**O**_**8**_ contains naphthalene and coumarin moieties, while **T** bears a pyrene group. The supramolecular complexed structure was verified by ^1^H NMR studies in solution and X-ray single crystal analysis in the solid state. The host-guest binding constant was determined by isothermal calorimetric (ITC) studies (2.31 × 10^3 ^M^−1^). Steady-state and time-resolved fluorescence measurements confirmed the process of cascade FRET from naphthalene to pyrene and then pyrene to coumarin groups. This work is a proof-of-concept example and represents an early case of realizing a sequential two-step FRET process in discrete supramolecular assemblies.

Some other macrocycles have also been used to construct sequential FRET systems. For example, *β*-CD can bind chemiluminescence reagents and fluorophores to form dynamic nanoassemblies, which can bring the included luminescent intermediate and fluorophores into close proximity and proper alignment. On this basis, Ma and co-workers achieved highly efficient cascade FRET in the dynamic nanoassemblies of *β*-CD, chemiluminescence reagents, and fluorophores in aqueous media^93^. Xing and co-workers realized an artificial LHS with a two-step sequential FRET based on host-guest interactions of cucurbit[7]uril^94^. In a follow-up work, they built a sequential FRET system based on supramolecular organic frameworks^95^. Recently, Hu and co-workers reported a supramolecular LHS with cascade energy transfer for photocatalysis based on cavitand (Fig. 3c)^96^. Resorcinarene-based cavitands are a class of macrocyclic compounds with deep cavities that have been widely used in supramolecular chemistry. In this work, the authors first synthesized a water-soluble cavitand (**H2**) and a TPE-derived diamantadine guest (**G2**). **H2** can complex well with **G2** to enhance its AIE behavior and further form nanoparticles in aqueous solution. By further co-assembly with **ESY** as the relay acceptor and **NiR** as the final acceptor, an efficient cascade FRET system was obtained, accompanied by a fluorescence change from light blue to green to red. The FRET efficiencies were determined to be 57% for the first step and 71% for the second step. Moreover, the antenna effect of the first step was calculated to be 25.8 ([**H2**⊃**G2**]/[**ESY**] = 200:1) and was 7.5 for the second step when [**H2**⊃**G2**]/[**ESY**]/[**NiR**] = 200 : 1 : 1. Interestingly, the obtained system showed excellent photocatalytic capabilities in cross-dehydrogenative coupling (CDC) reaction. This study not only expands the applications of cavitands but also provides a new paradigm for mimicking photosynthesis in nature, which may inspire new idea for photocatalysis by using host-guest assemblies.

## Multi-step FRET systems constructed by metal-coordination scaffolds

In recent years, the combination of discrete non-covalent coordination complexes (e.g., metallacycles and metallacages) and fluorophores gave rise to novel types of fluorescent metal-coordination scaffolds, which were further used for the construction of energy transfer systems. As early as in 2005, Würthner and co-workers reported an artificial LHS with one-step FRET based on a coordinated molecular square^97^. After that, a number of one-step energy transfer systems driven by metal-coordination interaction have been fabricated by introducing energy donors and acceptors into the scaffolds^98–102^. It is worth mentioning that the orthogonal self-assembly of metal coordination and π-π interaction or host-guest interaction provides further opportunities for constructing multi-functional FRET systems. In the past three years, two-step FRET or even three-step energy transfer systems based on metal coordination have been reported. In this section, we focus on these multi-step FRET systems constructed from metal-coordinated scaffolds.

**Fig. 4:**
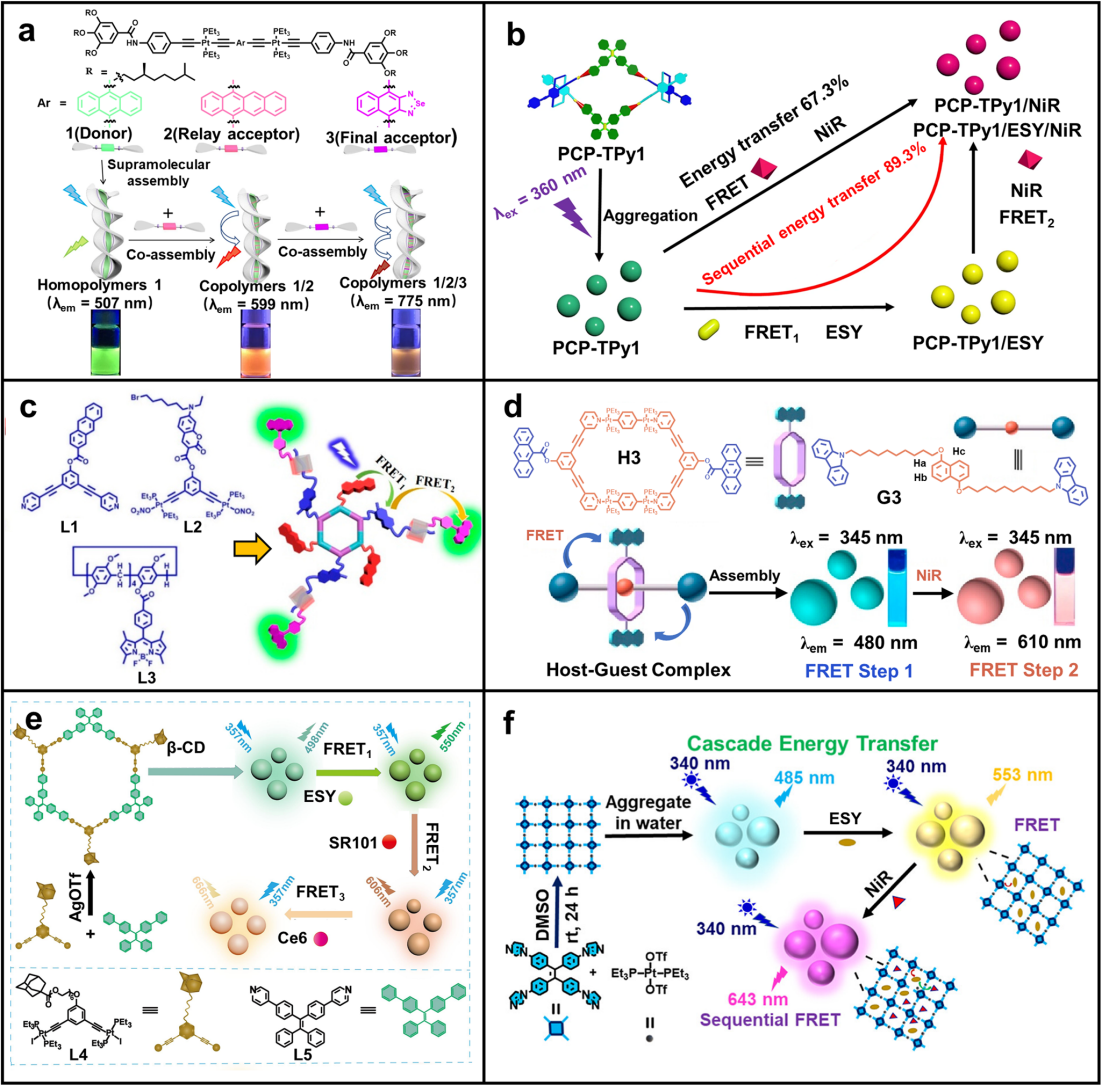
Multi-step FRET systems constructed by metal-coordination interactions. **a** Supramolecular copolymerization of **1,** **2**, and **3** with the sequential FRET behaviors. Reprinted with permission from ref. ^103^, Copyright 2022 Nature Publishing Group. **b** Construction of artificial LHSs based on double helicate **PCP**-**TPy1**. Reprinted with permission from ref. ^104^, Copyright 2023 Nature Publishing Group. **c** Cartoon representation of the cascade FRET system constructed by orthogonal self-assembly. Reprinted with permission from ref. ^18^, Copyright 2021 American Chemical Society. **d** Illustration of the two-step FRET system based on metallacycle **H3** and guest molecule **G3**. Reprinted with permission from ref. ^106^, Copyright 2023 American Chemical Society. **e** Representation of the three-step FRET system. Reprinted with permission from ref. ^108^, Copyright 2023 Elsevier. **f** Schematic illustration of supramolecular coordination polymer-based LHS with sequential energy transfer. Reprinted with permission from ref. ^109^, Copyright 2022 American Chemical Society.

Most photosynthetic organisms utilize rigid protein scaffolds to bind pigments and control their energy transfer. However, the overall energy transfer efficiency of most artificial systems is lower than that of purple photosynthetic bacteria (almost 100%). This may be due to the irregular arrangement of donor/acceptor (D/A) in these artificial scaffolds. Inspired by the dense bacteriochlorophyll (BChl) pigments in green photosynthetic bacteria, Wang and co-workers prepared a two-step FRET system with high energy transfer efficiency through π-π stacking interaction of three different σ-platinum (hetero)acenes^103^. As shown in Fig. 4a, these building blocks of **1** can stack with two types of acceptor monomers (**2** and **3**) to form supramolecular copolymers, which show high exciton migration rates and long transfer distance of excitation energy. As a result, the overall energy transfer efficiency reached 87.4%. This work exploits carefully designed supramolecular strategies to achieve nanoscale ordered structures, thereby successfully achieving efficient cascade energy transfer. In another work, Jiang and co-workers reported an artificial LHS with two-step FRET by utilizing [2,2] paracyclophanes-based double helicates as scaffold (Fig. 4b)^104^. These double helicates showed significant AIE effect in THF/H_2_O (v/v = 1:9) solvent and thus could be served as energy donors. By using **ESY** and **NiR** as energy acceptors, the aggregated double helicates can form a sequential two-step LHS with the energy transfer efficiency up to 89.3%. Finally, white light-emitting devices were achieved by doping energy-transfer materials into PMMA films and coating them on blue LED bulbs.

By adopting an orthogonal self-assembly approach, Yang and co-workers prepared a two-step FRET system through platinum-based coordination interactions and pillararene-based macrocyclic host-guest interactions (Fig. 4c)^18^. In this system, a series of fluorophores, such as anthracene, coumarin, and BODIPY, were well arranged in the scaffold at precise distances, which enables efficient two-step energy transfer. As a result, the ^1^O_2_ generation efficiency of this sequential FRET system is 1.5 times higher and the photooxidation activity is 1.2 times higher than that of the corresponding one-step system. This work not only demonstrates an efficient multi-step FRET system through orthogonal self-assembly but also provides new ideas for creating smart multi-responsive materials. In another work, Shi and co-workers reported a two-step FRET system by using platinum metallacycle based rotaxanes as energy donor and **ESY** and **NiR** as acceptors^105^. Later, they introduced chromophores onto both the metallacycles and dumbbells (Fig. 4d)^106^. Multiple chromophores including anthracene and carbazole were easily incorporated into the metallacycles and the guest, respectively. This strategy brings the chromophores into close proximity, facilitating an efficient FRET process. The obtained two-step FRET system was further used to prepare white light-emitting diodes and used as a nanoreactor for photocatalytic reactions.

In 2021, Zhang and co-workers reported a sequential energy transfer system based on metallacycle, which was used to catalyze the alkylation of C–H bonds in aqueous solution^107^. In a follow-up work, they further developed a three-step FRET system based on metallacycles (Fig. 4e)^108^. In this system, the AIE-active metallacycles was used as energy donor/antenna, **ESY** and **SR101** served as conveyors, and near-infrared emissive chlorin-e6 (**Ce6**) was used as the final energy acceptor. The donors and acceptors were co-assembled by non-covalent interactions. The metallacycle was constructed from **L4** and **L5** through silver coordination. *β*-CD was introduced by host-guest interaction to enhance the hydrophilicity of the assemblies, which ensured that the donor and acceptor were in close proximity to each other for an efficient FRET. In another work, Mukherjee and co-workers constructed TPE-based emissive Pt(II) coordination polymers toward artificial LHSs with cascade energy transfer (Fig. 4f)^109^. The coordination polymer exhibited significant emission enhancement in water/DMSO (v/v = 9/1) mixture as it further self-assembled into discrete spherical nanoparticles. The hydrophobic cavities can play the role of a suitable host to encapsulate organic dyes, such as **ESY** and **NiR**, to achieve efficient cascade FRET.

## Multi-step FRET systems constructed by biomolecular scaffolds

In natural light-harvesting systems, antenna chromophores are tightly packed around protein scaffolds. They absorb solar energy and transfer it to acceptors, and ultimately to the reaction center for conversion into chemical energy. Inspired by nature, supramolecular chemists have been trying to use biomolecular scaffolds such as DNA and peptides/proteins to construct artificial LHS based on FRET^110–112^. In 2021, Perrier and co-workers reported an efficient LHS with two-step FRET based on cyclic-peptide (**CP**) nanotubes in aqueous media^113^. The authors synthesized three chromophore attached cyclic-peptide monomers with poly-(ethylene glycol) (PEG) tails: cyanine3-CP-PEG (**Cy3-CP-PEG**), naphthalene monoimide-CP-PEG (**NTI-CP-PEG**), and pyrene-CP-PEG (**PYR-CP-PEG**). These cyclic monomers can self-assemble into nanotubes through non-covalent interactions in water. As a result, efficient sequential FRET can take place from **PYR-CP-PEG** to **Cy3-CP-PEG** through **NTI-CP-PEG**, showing a FRET efficiency up to 95%. At the same time, the fluorescence color of the solution could be tuned from blue to green to orange. Notably, the ACQ effect of the chromophores was greatly suppressed due to their slipped stacking arrangement along the nanotubes. Their findings provide a general approach to design efficient cascade FRET systems based on peptides and build strongly emissive organic materials in water.

**Fig. 5:**
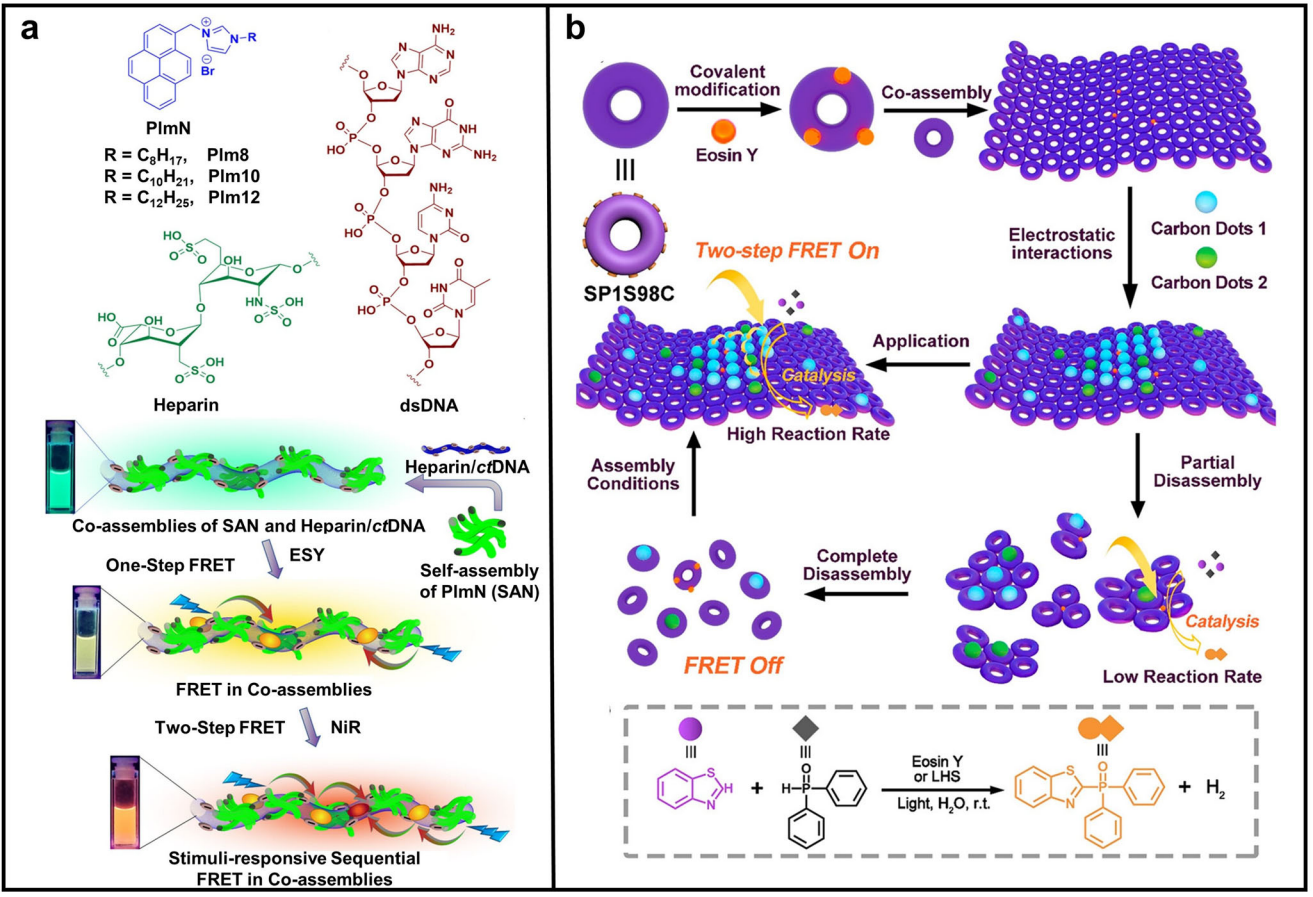
Multi-step FRET systems constructed by biomolecular scaffold. **a** Upper: chemical structures of **PImN**, heparin, and dsDNA; down: schematic representation of the stimuli-responsive cascade energy transfer in co-assemblies. Reprinted with permission from ref. ^114^, Copyright 2023 American Chemical Society. **b** Schematic representation of the construction of the LHS with two-step FRET and the control of the “On/Off” state of the FRET process. Reprinted with permission from ref. ^115^, Copyright 2022 American Chemical Society.

Tightly packed conventional fluorophores with flat structure (e.g., pyrene) often undergo severe self-quenching. Recently, Banerjee and co-workers reported a cascade FRET system based on co-assembly of cationic pyrene derivatives and two anionic biomolecular scaffolds (DNA and heparin) in aqueous solution (Fig. 5a)^114^. Specifically, the authors first synthesized a series of cationic pyrene appended imidazolium salt (**PImN**) as energy donor. They are amphiphilic and can self-assemble into nanoaggregates in water, which are designated as **SAN**. They further complexed with anionic DNA and heparin to serve as excellent platforms for constructing cascade FRET systems upon including external dyes, such as **ESY** and **NiR**. Energy transfer efficiency was up to 90% for the single step and ∼60-70% for the two-step FRET. As a result, multi-color emissive materials with tunable properties were prepared both in solution and polymer films. More interestingly, the authors found that the system is pH and temperature dependent. On this basis, they applied these materials in ratiometric temperature sensing and information encryption.

In order to mimic the natural light-harvesting system in both structure and function, Sun, Liu and co-workers reported an “On/Off” switchable cascade FRET system based on single-layered protein nanosheets formed from cricoid stable protein one (**SP1**)^115^. As shown in Fig. 5b, the wheel-shaped **SP1** can be transformed into **SP1S98C** variant with 12 cysteines, which could further form 2D nanosheet structure through dynamic covalent bond. They utilized two kinds of nontoxic amino-abundant carbon dots, **CD1** and **CD2**, as energy donor and acceptor, respectively, due to their well-matched photophysical properties. These carbon dots could be introduced into the protein nanosheets through electrostatic interactions. Moreover, **ESY** was further incorporated into the scaffolds to serve as the second acceptor to harvest excitation energy from **CD2**. The energy collected by **ESY** was further applied to catalyze a cross-coupling hydrogen evolution reaction. Interestingly, this sequential FRET process could be controlled by redox agents, resulting in an “On/Off” switching of the final product yield. This work not only provides a typical method for the fabrication of biomimetic photosystems based on protein self-assembly, but also provides promising insights into the development of artificial photosynthetic systems.

## Summary and outlook

In conclusion, recent advancements of multi-step FRET systems based on discrete supramolecular assemblies are summarized in this perspective. A diverse range of non-covalent scaffolds, such as amphiphile-based nanoparticles, host-guest complexes/nanoaggregates, metal-coordination scaffolds, and biomolecular scaffolds, have been employed to support multiple chromophores to achieve multi-step FRET. These chromophores are brought into close proximity through non-covalent interactions, greatly reducing the tedious synthesis of covalent systems and improving the FRET efficiency. To avoid fluorescence quenching in these discrete supramolecular assemblies, AIE-type energy donors/antennas are used in many cases. At the same time, the photophysical properties of the donor and acceptor need to be well matched. These constructed systems have a wide range of applications, from tunable photoluminescent materials to information encryption materials and photocatalysts.

Although supramolecular cascade FRET systems have made a lot of progress, it still need to solve the following challenges in future development: (1) The current selection of energy acceptors is very narrow, and many examples use **ESY** and **NiR** as the first and secondary acceptor, however, in order to meet the needs of a wider range of applications, more matching acceptors need to be developed and found. (2) Most of the examples described in this perspective are two-step FRET systems, and there is only one example of a three-step FRET system^108^. The construction of a three-step FRET system is more challenging and is also one of the directions for future efforts. (3) The stability of these fluorescent systems has rarely been considered. However, in order to meet the requirements of practical applications, it is necessary to construct sequential FRET systems with long-term stability. (4) Although these systems have demonstrated some preliminary applications, how to push them into valuable practical applications still requires a lot of effort. In summary, there is still a lot of room for further development in this field, and we believe it will have a bright future, and more functional multi-step FRET systems will be created.
